# Effect of Passage Number and Culture Time on the Expression and Activity of Insulin-Degrading Enzyme in Caco-2 Cells

**DOI:** 10.22034/ibj.22.1.70

**Published:** 2018-01

**Authors:** Taiebeh Mohammadi Farsani, Elahe Motevaseli, Nadia Neyazi, Mohammad Reza Khorramizadeh, Elaheh Zafarvahedian, Mohammad Hossein Ghahremani

**Affiliations:** 1 Department of Medical Biotechnology, School of Advanced Technologies in Medicine, Tehran University of Medical Sciences, Tehran, Iran; 2 Department of Molecular Medicine, School of Advanced Technologies in Medicine, Tehran University of Medical Sciences, Tehran, Iran; 3 Department of Medical Biotechnology, School of Advanced Technologies in Medicine, Tehran University of Medical Sciences, International Campus (TUMS- IC), 88 Italia St., Tehran, Iran; 4 Biosensor Research Center, Endocrinology and Metabolism Molecular-Cellular Sciences Institute, Tehran University of Medical Sciences, Tehran, Iran; 5 Department of Pharmacology and Toxicology, Faculty of pharmacy, Tehran University of Medical Sciences, Tehran, Iran

**Keywords:** Metalloendopeptidase, Alzheimer’s disease, Caco-2 cells

## Abstract

**Background:**

Insulin-degrading enzyme (IDE) is a conserved zinc metallopeptidase. Here, we have evaluated the effect of passage number and culture time on IDE expression and activity in colorectal adenocarcinoma cell line (Caco-2).

**Methods:**

Caco-2 cells were cultured with different passage ranges of 5-15, 25-35, 52-63 for 48, 72, and 120 hours. Subsequently, IDE expression and enzyme activity were assessed by Western blot analysis and fluorescent assay, respectively.

**Results:**

Our results confirmed that the amount of IDE was higher in cell extract compared to supernatant, and different passage numbers and culture times had small effect on IDE expression. However, when cells were cultured in the passage number range of 5-15 for 72 hours, the IDE activity was 35% higher compared to other passage numbers (*p* < 0.05).

**Conclusion:**

The use of Caco-2 cells at passage number range of 5-15 and culture time of 72 hours provides proper conditions for IDE-related studies.

## INTRODUCTION

Insulin-degrading enzyme (IDE) is a highly conserved zinc metallopeptidase initially described for its ability to degrade insulin, β-amyloid, glucagon, amylin, somatostatin, and natriuretic peptide[1]. Previous studies have linked IDE to the etiology of diseases, such as Alzheimer’s disease and insulin-dependent diabetes mellitus[2-4]. Moreover, it has been shown that following different stresses on normal and malignant cells, the IDE levels will be upregulated similar to a heat shock protein[5]. Therefore, the IDE modulators can be helpful in treating diseases such as Alzheimer’s disease and insulin-dependent diabetes mellitus[6]. Thus, introducing a suitable *in vitro* cell source will be very important to study IDE and to explore its modulators and related drugs.

Human colorectal adenocarcinoma cells (Caco-2 cells) have been used in *in vitro* studies for more than 30 years[7]. It has been reported that IDE in these cells is responsible for the majority of insulin clearance[8-10]. Although IDE has predominant presence in the cytosol, it is also found in subcellular compartments[11,12]. In *in vitro* studies, there are inter-laboratories variability produced by the cell culture-related factors such as passage number, age in culture[12], serum and supplements, and the source of the clones[13,14]. Cell lines with higher passage numbers exhibit alterations in cells morphology and functions[12]. Similarly, in Caco-2 cell, some factors such as passage number, composition of the medium, and the culturing system can affect its proliferation, differentiation, and physiological properties[12-16]. Since Caco-2 cells are being used as the IDE source, in this study, we have evaluated the IDE enzyme level and enzyme activity in various passage numbers. Caco-2 cells have been used in various passage numbers from <25 to ≥100[17,18]. A recent report has shown that sucrase-isomaltase activity is maximized in cells beyond passage 100[19]. Overall, it is recommended that the cells are used within a relatively limited range of passages for which the cells properties have been well-characterized[20].

So far, there is no study to focus on the effect of different passage numbers and the age in culture in Caco-2 cell lines on IDE quantity or activity. Therefore, we tested the IDE level and its activity in Caco-2 cell regarding different passage numbers and the age in culture.

## MATERIALS AND METHODS

RPMI 1640 and penicillin-streptomycin were provided from Biosera (England). DMEM-F12, FBS, Trypsin-EDTA were purchased from PAA Laboratories (Austria). Trypan blue (0.4% w/v) and MTT were from Sigma (USA). Western blot chemiluminescence detection kit (Roche, Germany) and polyvinylidene difluoride membrane were obtained from Roche Applied Science (Germany). IDE and β-actin antibodies were procured from Cell Signaling Technology (USA), secondary horseradish peroxidase (HRP)-conjugated antibody from Bio-Rad (USA), and substrate V from R&D systems (Minneapolis, USA). All other chemicals were purchased from Merck Chemical Co. (Germany). Caco-2 cells were obtained from National Cell Bank of Iran (Pasteur Institute of Iran, Tehran).

### Cell culture

Caco-2 cells (passage number 3) were grown in 50% RPMI 1640, 34% DMEM-F12, 15% FBS, and 1% penicillin-streptomycin in a 25-cm^2^ plastic flask in humidified atmosphere of 5% CO_2_ at 37 °C for 48, 72, and 120 hours. Culture medium was changed every second day. The cells were trypsinized after reaching the 70-80% confluency. For various passages, using a standard procedure, the cells were passaged in three different ranges (5-15, 25-35, and 52-63). The passage range of 5-15 was selected as the earliest range that was used in our experiments, 25-35 was used based on previous studies[14,21], and 52-63 was assayed because it was known to affect the protein expression, and it was the oldest passage available at the time of experiments.

### Cell harvest

Cell extracts and supernatants were collected at 48, 72, and 120 hours after seeding. Cells extracts were harvested by lysing the cells with Triton X-100 (1% in Tris-buffered saline [TBS]; 12 h, 4 °C) and subsequent centrifugation (11520 ×g, 10 min, 4 °C). Cell extracts and supernatants were transferred to a clean pre-cooled tube and stored at -80 °C for further analysis. On the day of experiment, the protein content of supernatant and cell extracts were determined by the BCA Protein Assay Kit (Parstous, Iran).

### Expression of insulin-degrading enzyme in Caco-2

An equal amount of samples (~50 µg) were mixed in a loading buffer (62.5 mM Tris HCl, pH 6.8, 50 mM dithiothreitol, 10% SDS, 10% glycerol, and 0.25% W/V Bromophenol blue), denatured by boiling for 10 min, subjected to a 10% SDS-PAGE and transferred onto PVDF membranes. The membranes were blocked using casein 1% in 0.1% Tween20 in TBS (TBST) at 4 °C for 12 hours and subsequently incubated with rabbit polyclonal anti-IDE antibody (1:5000) or mouse anti β-actin antibody (1:5000, as an internal control) at 4 °C overnight. Blots were then incubated by secondary HRP-conjugated antibody (1:5000) at room temperature for 1 hour and detected using chemiluminescence (Roche, Germany) on photodoc.

### Insulin-degrading enzyme activity assessment

The IDE activity was assessed in triplicate in 100 µl reaction, including 1 µM substrate V, 50 µl reaction buffer (10 µM ZnCl_2_, 50 mM NaCl, and 100 mM Tris-Cl, pH 7.5) and 40 µl (250 µg) of supernatant or cell extract. The hydrolysis of substrate V was monitored on a Synergy 4 microplate reader (Biotek, USA) with excitation and emission wavelengths set at 327 and 395 nm, respectively. The IDE activity was measured at 15 min and calculated as relative fluorescent unit (RFU) per loaded protein.

### Statistical analysis

The results were expressed as mean ± SEM. of three independent experiments. The data were analyzed by two-way ANOVA, followed by Bonferroni comparison using Graph Pad Prism 5 (San Diego, USA). *p* < 0.05 was considered statistically significant.

## RESULTS

### Insulin-degrading enzyme expression in cell extract and supernatant of Caco-2

**Fig. 1:**
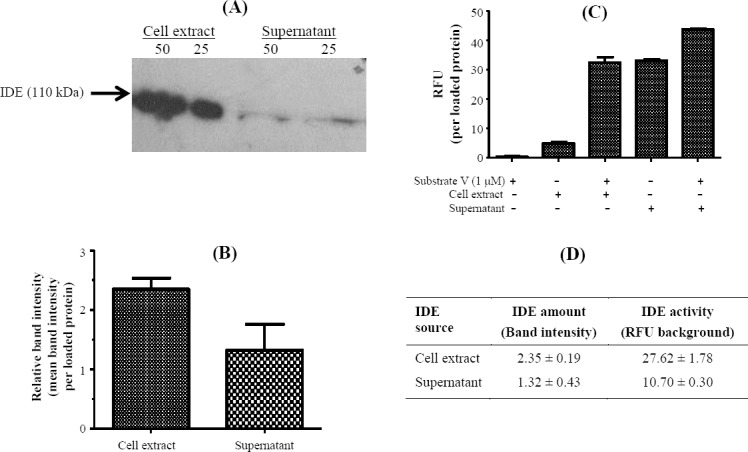
Protein expression and the activity of insulin-degrading enzyme (IDE) analysis by Western blot and fluorescent assay, respectively. (A) Caco-2 cells were cultured, and an equal volume of supernatant and cell extract was analyzed by Western blot using anti-IDE antibody. β-actin was used as the internal control, (B) the detected bands were digitized, and the intensity was calculated as relative intensity to loaded protein of cell extract and supernatant samples, (C) an equal volume of supernatant and cell extract were analyzed by fluorescent assay using substrate V. The relative fluorescent unit (RFU) of samples was calculated as RFU per loaded protein of cell extract and supernatant samples (RFU/loaded protein), (D) Comparison between insulin degrading enzyme (IDE) amount (band intensity/loaded protein) and activity (RFU-Background) in the cell extract and cell supernatant.

The level of IDE in Caco-2 was compared using Western blot, and the relative band intensity was calculated (Fig. 1A and 1B). The mean IDE expression was 2.34 ± 0.19 and 1.32 ± 0.43 in cell extract and supernatant, respectively. Thus, the expression of IDE in cell extract was 43.61% more than the supernatant.

### Insulin-degrading enzyme activity in cell extract and supernatant of Caco-2

For IDE activity, we measured the enzyme activity in cell extract and supernatant of Caco-2 cells. The enzyme activity was calculated as RFU per loaded protein and subtracted from background (Fig. 1C). The results showed the IDE activity in cell extract samples were 27.62 ± 1.78 and 10.70 ± 0.30 RFU, respectively (Fig. 1C). Therefore, the IDE activity in the cell extract was 61.25% more than supernatant (Fig. 1D). It should be mentioned that the supernatant had fairly high background fluorescence (Fig. 1C), which is mainly due to fluorescent absorbance of FBS[22].

### Insulin-degrading enzyme expression in three different passage ranges and times of cell culture

**Fig. 2:**
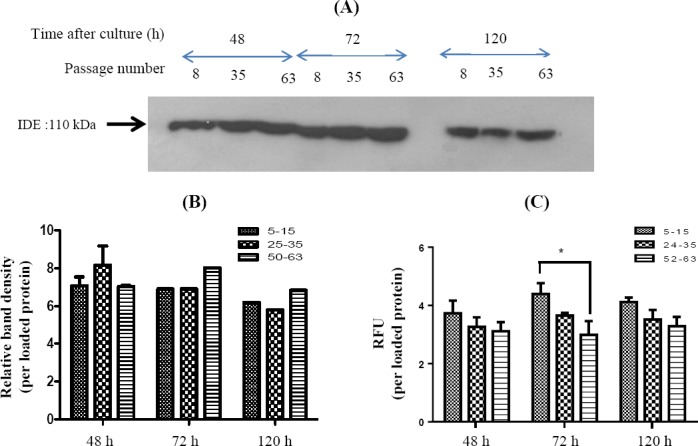
Protein expression and the activity of insulin-degrading enzyme (IDE) were analyzed in different passages of Caco-2 cell extracts. (A) Three different passage ranges of Caco-2 cells were studied in three different time points. Cells were cultured, and an equal volume of cell extracts were analyzed by Western blot using anti-IDE antibody. β-actin was used as the internal control, (B) the detected bands were digitized, and the intensity was calculated as relative intensity to loaded protein of cell extract, (C) an equal volume of samples was analyzed by fluorescent assay using substrate V. The relative fluorescent unit (RFU) of samples was calculated as RFU per loaded protein of cell extract (*p < 0.05, n = 3)

The IDE expression in three different passage ranges and three culture times of Caco-2 cell (Fig. 2A) showed that the IDE protein level in cell extract was not significantly different in various passage numbers and culture times (*p* = 0.72 and *p* = 0.33, respectively, Fig. 2B).

### Insulin-degrading enzyme activity in different passage numbers and different times of cell culture

The IDE activity was determined in various passage number ranges, and RFU per loaded protein was calculated (Fig. 2C). The results showed that in passage number ranges of 24-35 and 52-63, the IDE activities were not significantly different in various time points (*p* = 0.12 and *p* = 0.24, respectively). However, in lower passage number (5-15), the data analysis showed that IDE activity was ~45% more than higher passage ranges (52-63) in 72-h culture time (Fig. 2C, *p* < 0.05). Therefore, the lower passage number and ~72 h after seeding will be the best time to use cell extract for IDE assay.

## DISCUSSION

Previous studies have reported Caco-2 cells as a source with suitable I DE expression and activity[9,10]. Therefore, Caco-2 cell line has been used as a source for IDE experiments such as the selection of new drug candidates including inhibitors and activators of IDE. Here, we tested the effect of cell passage number on the IDE expression and activity with regard to passage number.

IDE is mainly a cytosolic protein and is secreted in a nonconventional pathway in association with exosome[23]. We found that the amount of IDE in the cell extract of Caco-2 cells was 43.61% more than supernatant. Furthermore, the IDE activity was higher in the cell extract (27.62 ± 1.78 RFU) than supernatant (10.70 ± 0.30 RFU). These results are in agreement with those of other researchers who have used the cell extract as the IDE source[8,10,24].

In many cases, variability in cell culture experiments has been linked to the passage number or senescence of cell line, as well as culture medium. Studies have shown the effect of passage number of Caco-2 on growth, viability, efflux, protein expression, and Transepithelial electrical resistance[12,14]. Therefore, an essential part of designing study protocols is to elucidate the effect of these factors on producing variable results. Based on our results, the IDE expression had small changes in different passage numbers (5-15, 25-35, and 52-63), and the activity was similar in higher passage numbers. These findings are similar to Briske-Anderson *et al*.[14] findings in which they have assessed the effect of passages 22, 33, 72 and days of development (0-30 day) on total protein in Caco-2 cells. They have also shown that the total cellular protein per membrane during 192 hours were similar among passages[14].

Despite small changes in IDE expression among different passage numbers, our data showed that the passage number 5-15 had higher IDE activity than others after 72-hour culture time (*p* < 0.05, Fig. 2C). It has been reported that the culture condition and passage number can influence the protein content and function of Caco-2 cells[21,25]. The effect of passage number depends on the type of protein as well. In a study on P-gp expression in Caco-2 cells, higher passage number (>40) is essential since P-gp is very low in passage less than 18[25]. However, in efflux experiments, the lower passage number (<40) is requiredbecause the efflux rate is decreased dramatically in the higher passage number[26]. Similarly, in Caco-2 cells, longer periods (6-15 days) of culture have reduced function of the ubiquitin degradation system[26]. Considering the role of IDE function in protein degradation system[5,27], one can conclude a lower activity of IDE in higher passage numbers. Our results indicate a lower IDE activity in the higher passage number similar to the findings of Zhang *et al*.[27]. Thus, for IDE activity experiments, the lower passage number and ~72-h culture have the highest IDE activity in Caco-2 cell extracts.

The present study demonstrates that the passage number or culture time can influence the IDE activity, but not the IDE expression. The IDE activity was higher in lower passage numbers (5-15) at 72-h culture. Collectively, Caco-2 cells in studies related to IDE should be used in lower passage numbers.
